# Supplementary material for: Neural circuits underlying auditory contrast gain control and their perceptual implications
Source: Nat Commun. 2020 Jan 16;11:324. doi: 10.1038/s41467-019-14163-5 (PMC6965083; doi:10.1038/s41467-019-14163-5)
Supplement: Supplementary file 3 — Reporting Summary [file 41467_2019_14163_MOESM3_ESM.pdf]

nature research

Corresponding author(s): Michael Lohse

Last updated by author(s): 2 Nov 2019

Reporting Summary

Nature Research wishes to improve the reproducibility of the work that we publish. This form provides structure for consistency and transparency in reporting. For further information on Nature Research policies, see Authors & Referees and the Editorial Policy Checklist.

Statistics

For all statistical analyses, confirm that the following items are present in the figure legend, table legend, main text, or Methods section.

n/a	Confirmed	
☐	☒	The exact sample size (*n*) for each experimental group/condition, given as a discrete number and unit of measurement
☐	☒	A statement on whether measurements were taken from distinct samples or whether the same sample was measured repeatedly
☐	☒	The statistical test(s) used AND whether they are one- or two-sided<br>*Only common tests should be described solely by name; describe more complex techniques in the Methods section.*
☐	☒	A description of all covariates tested
☐	☒	A description of any assumptions or corrections, such as tests of normality and adjustment for multiple comparisons
☐	☒	A full description of the statistical parameters including central tendency (e.g. means) or other basic estimates (e.g. regression coefficient) AND variation (e.g. standard deviation) or associated estimates of uncertainty (e.g. confidence intervals)
☐	☒	For null hypothesis testing, the test statistic (e.g. *F*, *t*, *r*) with confidence intervals, effect sizes, degrees of freedom and *P* value noted<br>*Give P values as exact values whenever suitable.*
☒	☐	For Bayesian analysis, information on the choice of priors and Markov chain Monte Carlo settings
☒	☐	For hierarchical and complex designs, identification of the appropriate level for tests and full reporting of outcomes
☒	☐	Estimates of effect sizes (e.g. Cohen's *d*, Pearson's *r*), indicating how they were calculated

Our web collection on statistics for biologists contains articles on many of the points above.

Software and code

Policy information about availability of computer code

Data collection	Data were collected and saved using custom-written Matlab code (https://github.com/beniamino38/benware).
Data analysis	Matlab code for executing linear-nonlinear models used in this paper can be found on https://github.com/beniamino38/benlib.

For manuscripts utilizing custom algorithms or software that are central to the research but not yet described in published literature, software must be made available to editors/reviewers. We strongly encourage code deposition in a community repository (e.g. GitHub). See the Nature Research guidelines for submitting code & software for further information.

Data

Policy information about availability of data

All manuscripts must include a data availability statement. This statement should provide the following information, where applicable:

- Accession codes, unique identifiers, or web links for publicly available datasets
- A list of figures that have associated raw data
- A description of any restrictions on data availability

The data used to prodce all figures are provided in the source data file. Raw electrophysiology and behavioural data are available upon request to, and will be fulfilled by, the lead contact (michael.lohse@dpag.ox.ac.uk).

Field-specific reporting

Please select the one below that is the best fit for your research. If you are not sure, read the appropriate sections before making your selection.

☒ Life sciences ☐ Behavioural & social sciences ☐ Ecological, evolutionary & environmental sciences

Life sciences study design

All studies must disclose on these points even when the disclosure is negative.

Sample size	Sample sizes were determined by power calculations based on effect sizes of 5-10%.
Data exclusions	In the electrophysiology, units were excluded from analysis based on criteria for signal:noise ratio and STRF prediction quality, as described in the manuscript. These criteria were chosen to include as many units as possible, while excluding those which did not reflect neuronal activity. No data were excluded from the behavioural study.
Replication	Each electrophysiological finding is based on repeated experiments using multiple neurons, mice and experimental procedures, as specified in the manuscript, and results were consistent across all of these. Similarly, behavioural results were consistent across human participants.
Randomization	Animals were randomly selected for experiments by animal care staff without knowledge of the experiments. In every analysis, all non-excluded neurons from all animals were assigned to all conditions, so no random assignment was required. Similarly, all human subjects contributed data to all conditions for the experiment they participated in.
Blinding	All animals and human participants contributed to all conditions for the experiment they participated in, so there was no group allocation at this stage. Analysis was not blinded, but was conducted identically by computer on data from each condition.

Reporting for specific materials, systems and methods

We require information from authors about some types of materials, experimental systems and methods used in many studies. Here, indicate whether each material, system or method listed is relevant to your study. If you are not sure if a list item applies to your research, read the appropriate section before selecting a response.

Materials & experimental systems

n/a	Involved in the study	
☒	☐	Antibodies
☒	☐	Eukaryotic cell lines
☒	☐	Palaeontology
☐	☒	Animals and other organisms
☐	☒	Human research participants
☒	☐	Clinical data

Methods

n/a	Involved in the study	
☒	☐	ChIP-seq
☒	☐	Flow cytometry
☒	☐	MRI-based neuroimaging

Animals and other organisms

Policy information about studies involving animals; ARRIVE guidelines recommended for reporting animal research

Laboratory animals	C57BL6/J (Envigo, UK); GAD2-IRES-cre (JAX); VGAT-ChR2-YFP (JAX); C57BL6/NTac.Cdh23 (MRC Harwell, UK)
Wild animals	No wild animals were involved in the study
Field-collected samples	No field-collected samples were involved in the study
Ethics oversight	Committee on Animal Care and Ethical Review at the University of Oxford; UK Home Office

Note that full information on the approval of the study protocol must also be provided in the manuscript.

Human research participants

Policy information about studies involving human research participants

Population characteristics	Eight (4 male, 4 female) for the main experiment, plus two additional participants (both male) for the level control experiment. Age range 18-30 years old.
Recruitment	Participants were selected according to availability and had no known sources of bias in their auditory abilities.
Ethics oversight	Inter-divisional Research Ethics Committee at the University of Oxford

Note that full information on the approval of the study protocol must also be provided in the manuscript.
